# A comprehensive MRI-based computational model of blood flow in compliant aorta using radial basis function interpolation

**DOI:** 10.1186/s12938-024-01251-x

**Published:** 2024-07-23

**Authors:** Romana Perinajová, Thijn van de Ven, Elise Roelse, Fei Xu, Joe Juffermans, Jos Westenberg, Hildo Lamb, Saša Kenjereš

**Affiliations:** 1 Department of Chemical Engineering, Faculty of Applied Sciences, Delft University of Technology, Delft, The Netherlands; 2 J.M. Burgerscentrum Research School for Fluid Mechanics, Delft, The Netherlands; 3 Department of Radiology, Leiden University Medical Center, Leiden, The Netherlands

**Keywords:** Aorta, Thoracic aortic aneurysm, Computational fluid dynamics, Wall motion, Radial basis function, 4D-flow MRI

## Abstract

**Background:**

Properly understanding the origin and progression of the thoracic aortic aneurysm (TAA) can help prevent its growth and rupture. For a better understanding of this pathogenesis, the aortic blood flow has to be studied and interpreted in great detail. We can obtain detailed aortic blood flow information using magnetic resonance imaging (MRI) based computational fluid dynamics (CFD) with a prescribed motion of the aortic wall.

**Methods:**

We performed two different types of simulations—static (rigid wall) and dynamic (moving wall) for healthy control and a patient with a TAA. For the latter, we have developed a novel morphing approach based on the radial basis function (RBF) interpolation of the segmented 4D-flow MRI geometries at different time instants. Additionally, we have applied reconstructed 4D-flow MRI velocity profiles at the inlet with an automatic registration protocol.

**Results:**

The simulated RBF-based movement of the aorta matched well with the original 4D-flow MRI geometries. The wall movement was most dominant in the ascending aorta, accompanied by the highest variation of the blood flow patterns. The resulting data indicated significant differences between the dynamic and static simulations, with a relative difference for the patient of 7.47±14.18% in time-averaged wall shear stress and 15.97±43.32% in the oscillatory shear index (for the whole domain).

**Conclusions:**

In conclusion, the RBF-based morphing approach proved to be numerically accurate and computationally efficient in capturing complex kinematics of the aorta, as validated by 4D-flow MRI. We recommend this approach for future use in MRI-based CFD simulations in broad population studies. Performing these would bring a better understanding of the onset and growth of TAA.

## Background

Rupture of thoracic aortic aneurysm (TAA) is an acute medical condition, with a fatality rate of almost 95% [1]. Because of the high fatality, properly diagnosing and treating this dangerous condition is of utmost importance. However, the conventional guidelines that focus on the diameter and growth rate of TAA were shown to be inadequate in many cases [2, 3]. This emphasizes the need for new biomarkers that aim for patient-specific prediction of TAA rupture and look beyond the analysis based solely on the aorta geometry [4]. The blood flow information must be assessed to establish new predicting biomarkers. Such information can be obtained from 4D flow magnetic resonance imaging (MRI); however, its spatial and temporal resolution is limited [5]. In recent years, the clinical image-based computational fluid dynamics (CFD) [6] was successfully applied to provide the patient-specific blood flow features in great detail, for example, flow in aorta [7–9] and TAA [10] as well as in wider population studies [11, 12]. However, one important aspect of modeling the aorta or TAA is often omitted in the literature—the movement of the aorta (i.e., aorta kinematic). Because of the beating heart during the cardiac cycle, the aortic root moves downwards during systole and returns to its original position during diastole. This movement was reported to be approximately nine millimeters in the downward direction [13] with a clockwise twist up to 20° [14]. Furthermore, the aortic compliance causes the wall to expand and contract radially during the cardiac cycle due to the changing transmural pressure gradient over time [15]. It was reported that changes in the thoracic aorta diameter were in the 1.7 to 3.6 mm range [16]. These combined effects of the aortic wall kinetics can significantly affect the blood flow simulations, and consequently, they should be included in the CFD simulation [17].

To model the blood vessel movement, two simulation strategies have been applied in previous studies in the literature: (i) the fully coupled fluid–structure interaction (FSI), and (ii) the predefined wall displacement. The FSI studies of aorta hemodynamics were applied in [17–21]. However, the FSI method for patient-specific situations suffers from numerous limitations. These include the lack of detailed information on the aortic wall properties (i.e., non-homogeneous thickness and elasticity), difficulties with the physiological boundary conditions (for example, pressure), as well as the quite intensive computational costs (for example, iterative pre-stressing procedure, fluid/structure mechanics coupling). The estimation of the aorta motion was the focus of several studies in the literature [22–24]. A simplified method for the aortic wall motion was proposed in [24, 25]. The developed moving-boundary method (MBM) tuned with the non-invasive clinical images (2D cine-MRI) provided a good agreement with the FSI results [24] as well as with the measurements in terms of the luminal cross-sectional area [25]. The MBM method was also less computationally expensive. However, while the MBM methods show excellent agreement with the measurements in terms of the change of luminal radius throughout the cardiac cycle, they cannot capture the rotational and/or longitudinal movement.

To overcome this, the mesh-morphing approach based on radial basis function (RBF) was proposed for mimicking the motion of biological tissue, utilizing one-way coupling. Unlike the previously discussed FSI and MBM, the present method directly enforces the movement based on imaging data and therefore has the potential to closely mimic the complete movement of the arterial wall, while also being numerically efficient. While the choice of one-way coupling limits the method in the applications that consider the future progress of diseases, it can be an excellent tool for accurately simulating the present flow in arteries. RBF was successfully implemented in mimicking the motion of the aortic valve [26], left ventricle with mitral valve [27], and thoracic aorta [28, 29]. In the case of RBF application in the aorta, Capellini et al. [28, 29] presented an approach where only the ascending thoracic aorta (excluding root) was considered dynamic, and the rest of the domain was assumed to be rigid. Additionally, only a simplified inlet velocity boundary condition was implemented. These assumptions bring considerable simplifications to the complexity of motion and flow in the aorta.

To bridge these simplifications, we propose a proof-of-concept approach for a 4D-flow MRI-based compliant model of the aorta. This approach will be evaluated for the healthy control subject and patient-specific aorta with a large root aneurysm. For both cases, the movement of the aorta and all inlet and outlet boundary conditions will be extracted from the corresponding 4D-flow MRI scans. The dynamic behavior of the aorta will be mimicked by a morphing approach utilizing the radial basis function (RBF) interpolation based on Xu and Kenjereš [27]. We adapt the method to account for the motion of the whole thoracic aorta. To define the motion, we utilize 4D-flow MRI data at several points of the cardiac cycle. In addition, we present an automatic registration protocol of the 4D-flow MRI-derived velocity profile at the inlet for the moving aorta.

This article first introduces our main findings within the "Results" section, followed by the "Discussion" of these findings, and "Conclusions". Finally, the last section of our article focuses on a detailed explanation of the "Methods" that are utilized for this study.

## Results

### MRI-based wall movement

**Fig. 1:**
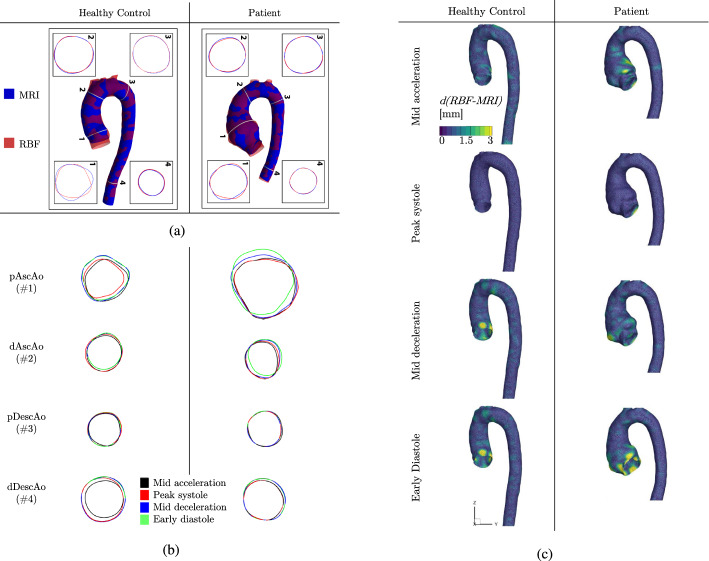
Comparison of the geometry for healthy control (HC) and patient (P) at mid acceleration for MRI (blue) and RBF (red) (**a**) for the whole aorta and cross-sections at proximal ascending aorta (pAscAo—1), distal ascending aorta (dAscAo—2), proximal descending aorta (pDescAo—3), and distal descending aorta (dDescAo—4); the evolution of RBF-based cross-sections at the key-frames (mid-acceleration—black, peak systole—red, mid-deceleration—blue, and early diastole—green) for the four locations (**b**); and the Euclidean distance between the RBF and MRI surface vertices ${\text{d(RBF-MRI)}}$ for HC and P at each key-frame **c**)

First, we want to assess the quality of the prescribed movement. The image-based movement of the aortic wall for both studied subjects: the healthy control (HC) and the patient with a TAA (P), is shown in Fig. 1. To validate the results of the RBF-based interpolation, we compare geometries extracted from the 4D-flow MRI (blue isosurface) and RBF-based reconstruction (red isosurface)—both at the mid-acceleration time instant; Fig. 1a. Furthermore, we also compare characteristic circumferential wall profiles at various cross-sections: (1) proximal ascending aorta (pAscAo); (2) distal ascending aorta (dAscAo), (3) proximal descending aorta (pDescAo), and (4) distal descending aorta (dDescAo), respectively. As can be seen in Fig. 1a, the RBF surface matches well the original 4D-flow MRI surface in the majority of the planes, except in the proximity of the root. Here, we can observe more variation. Additionally, in Fig. 1b, we show the time-evolution of the RBF-based circumferential profiles in identical cross-sections (i.e., planes (1–4)) at the four key-frames (black—mid-acceleration, red—peak systole, blue—mid-deceleration, green—early diastole). These circumferential profiles visualize local change in the area during the dynamic simulations, which is most pronounced close to the aortic root. Finally, the profiles in Fig. 1 give us only a qualitative understanding of local variation between RBF and 4D-flow MRI surfaces. Therefore, to see the variation for the whole surface, we have calculated the absolute Euclidean distance between each point of RBF-generated surfaces and the MRI segmentations. These data are shown for HC and P (at each key-frame) in Fig. 1c, and the median, mean, and standard deviation of the whole domain are reported in Table 1. Fig. 1c again showcases that the agreement between RBF and MRI surfaces is overall good, except in the proximity of the root. In addition, we can also observe an increasing variation in the agreement further from the peak systole.

**Table 1:** Median, mean, and standard deviation (std) for the absolute difference between RBF and MRI surface vertices (based on Fig. 1c) for healthy control (HC) and patient (P) at four key-frames (KF): KF1—mid-acceleration, KF2—peak systole, KF3—mid-deceleration, and KF4—early diastole

			KF 1	KF 2	KF 3	KF 4
HC	Median	[mm]	0.63	0.46	0.62	0.62
	Mean		0.69	0.45	0.70	0.70
	Std		0.35	0.18	0.41	0.43
P	Median	[mm]	0.57	0.46	0.59	0.63
	Mean		0.63	0.46	0.70	0.85
	Std		0.36	0.20	0.43	0.72

**Fig. 2:**
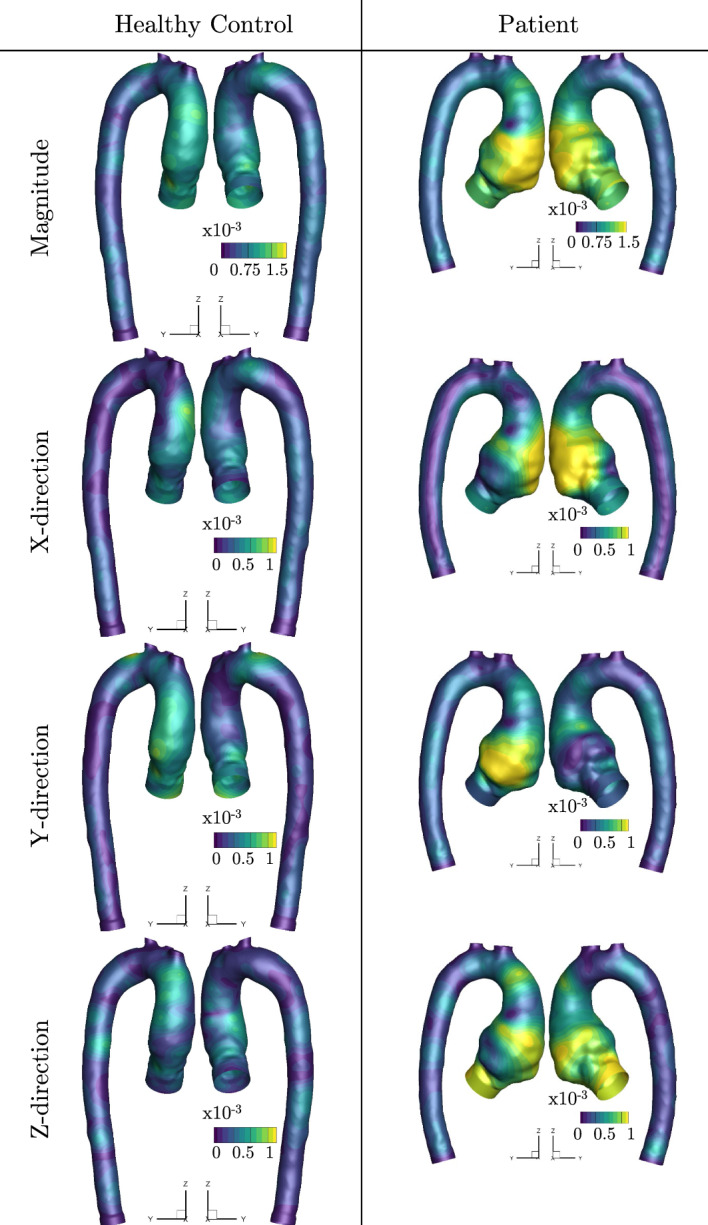
The time-average displacement (magnitude, *x*-direction, *y*-direction, and *z*-direction) during the cycle

To quantify the level of the aorta movement, the normalized time-averaged aortic wall displacements (magnitude and corresponding coordinate directions) are shown in Fig. 2. The normalization was done using the radius of the inlet plane (i.e., $r_{\text{HC}}^{\text{in}} = 1.49\cdot 10^{-2}$ m, and $r_\text{P}^\text{in} = 1.58\cdot 10^{-2}$ m). The simulated displacement is lower for healthy control in comparison to the patient. In addition, we can observe a clear higher displacement in the ascending aorta for both studied cases.

### Computational time

To compare the effect of the wall motion on the computational time for three simulated cycles, we report the wall time for one of the cases (HC). Both static and dynamic simulations (for this comparison) were run on 16 processors of AMD Opteron 6234. The reported wall time for static simulation was 33:07:59 (in [h:min:s]), and for the dynamic simulations, 38:29:31. The main differences in the computational time were related to the I/O intensive tasks (reading the prescribed mesh), rather than the simulations themselves.

### Effect of wall movement on blood flow

**Fig. 3:**
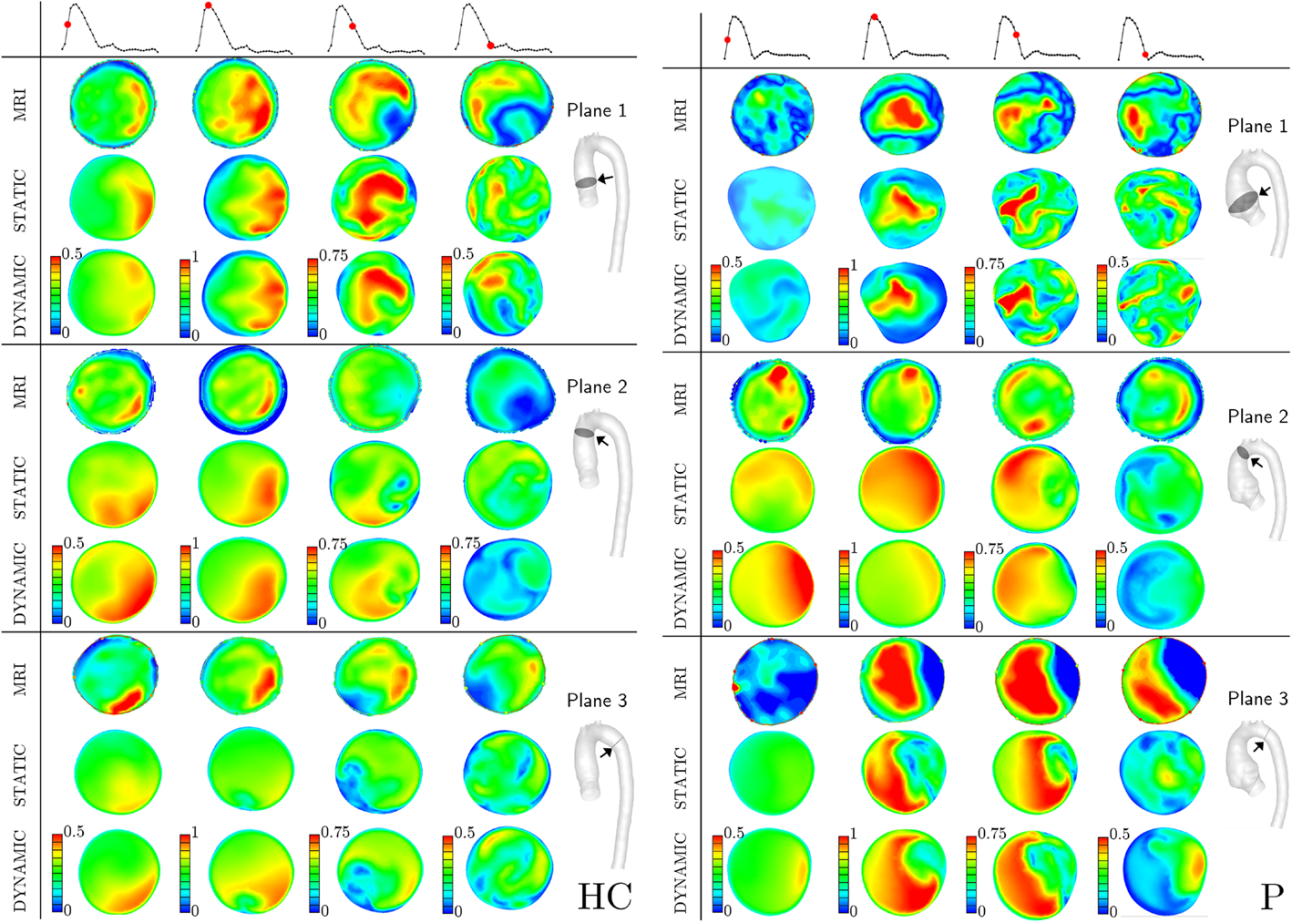
Velocity magnitude [m/s] at the visualized cross-sections of interest (proximal ascending aorta (pAscAo—1), distal ascending aorta (dAscAo—2), proximal descending aorta (pDescAo—3)) based on MRI, static, and dynamic CFD for healthy control (HC—left) and patient (P—right) at mid-acceleration, peak systole, mid-deceleration, and early diastole; the scale of velocity magnitude is adjusted per plane/time-step

Contours of the velocity magnitude at the pre-selected cross-sections (dAscAo, pAscAo, and pDescAo) at four time instants of the cardiac cycle (mid-acceleration, peak systole, mid deceleration, and early diastole), for the healthy and patient-specific cases (both with static and dynamic simulations) are shown in Fig. 3. For MRI, the velocity field was reconstructed from 4D-flow MRI data extracted in planes in the flow direction. Note that for better visualization, used color maps are specifically adjusted for different cross-sections and time steps. Overall, the computed profiles for HC resemble well the ones obtained by MRI, more differences can be observed for P, especially in planes 2 and 3. In addition, the results demonstrate a clear influence of movement on the calculated flow field, which is more significant for P.

### Effect of wall movement on $\mathrm {TAWSS}$ and $\mathrm {OSI}$  

**Fig. 4:**
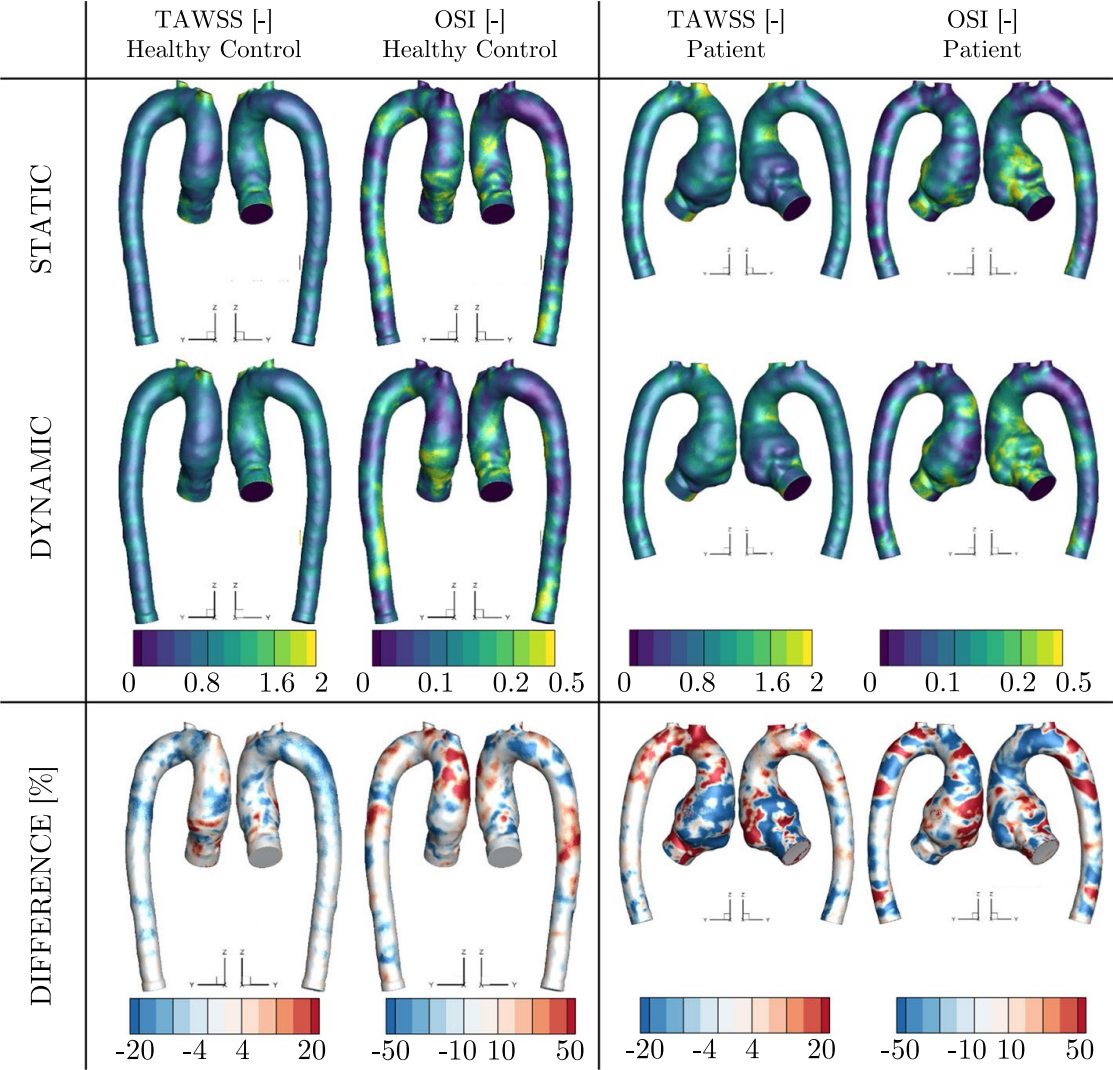
Time-averaged wall shear stress (TAWSS [Pa]) and oscillatory shear index (OSI [-]) based on static and dynamic simulations and the absolute percentage difference for the respective quantities between static and dynamic simulations for healthy control and patient

Time-averaged wall shear stress (TAWSS) and oscillatory shear index (OSI) are two important flow-derived quantities often mentioned in the literature as potential biomarkers to evaluate the progression of aortic aneurysms. The contours of the $\mathrm {TAWSS}$ and $\mathrm{ OSI}$ for the CFD_static_ and CFD_dynamic_ simulations are shown in Fig. 4. To make the comparison between the static and dynamic simulations easier, we also provided contours of the percentage differences $\Delta \mathrm {TAWSS}$ and $\Delta \mathrm {OSI}$. Note that for the dynamic simulation, contours of the TAWSS and OSI are shown for the peak-systole geometry. As can be seen for both subjects, we can observe slight differences between static and dynamic simulations for TAWSS, especially close to the root. For P, the differences in TAWSS are more pronounced in the whole ascending aorta. Dynamic simulations show significantly more differences for OSI; in both cases the differences in this quantity are more pronounced over the whole surface.

Next, we have also calculated the mean values of the absolute difference over the whole aorta surface (without side branches) for TAWSS and OSI. The mean value of absolute $\Delta \mathrm {TAWSS}$ for the healthy control was $\Delta \mathrm {TAWSS}_{\text {HC}}=2.72\pm 4.93\%$ Pa, and $\Delta \mathrm {TAWSS}_{P}=7.47\pm 14.18\%$ Pa for the patient-specific geometry. For the OSI difference, the mean value (of absolute $\Delta \mathrm {OSI}$ ) was $\Delta \mathrm {OSI}_{\text{HC}}=12.87\pm 43.92\%$ for the former, and $\Delta \mathrm {OSI}_{P}=15.97\pm 43.32\%$ for the latter.

**Fig. 5:**
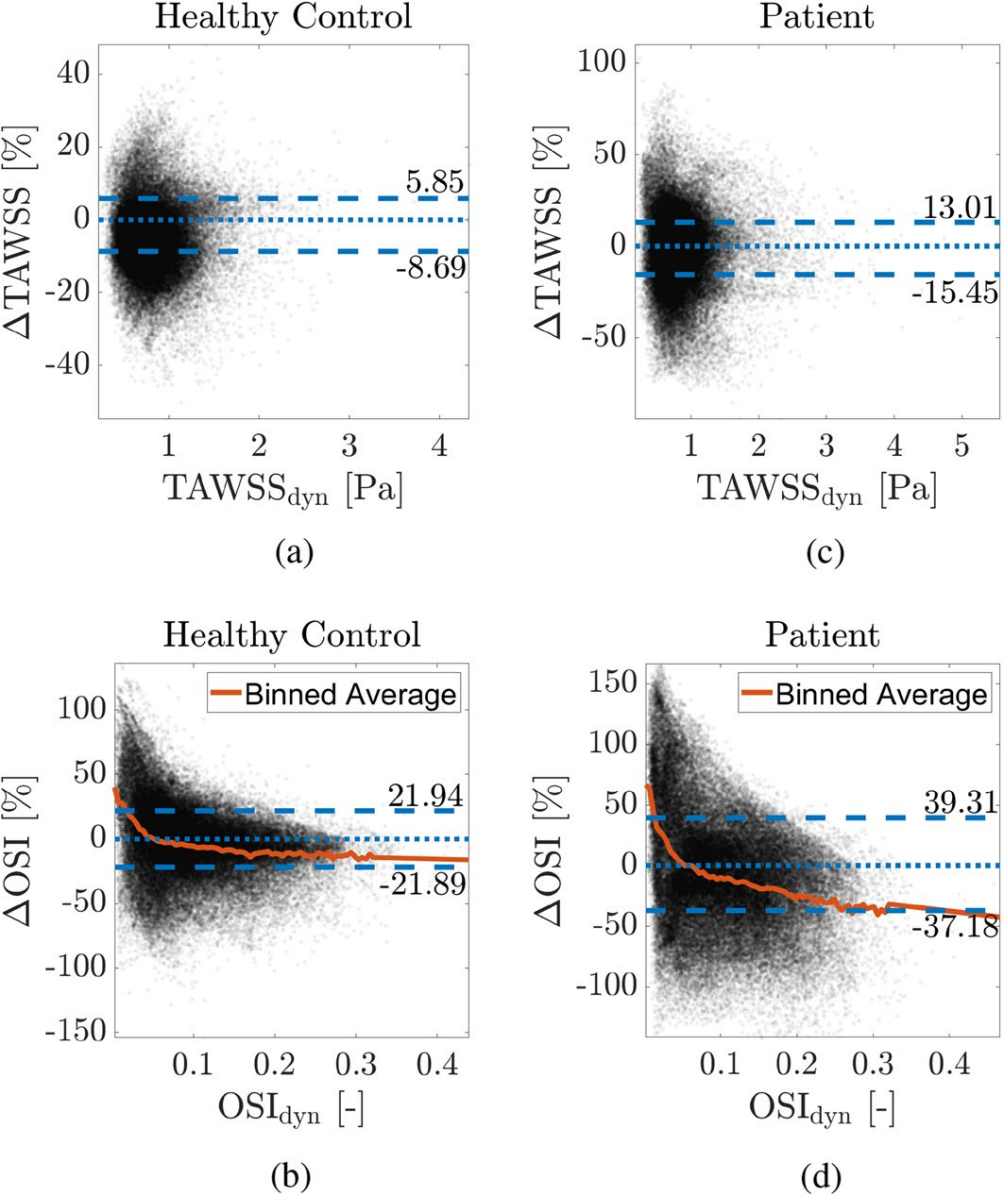
Scatter plots showcasing the time-averaged wall shear stress ($\mathrm {TAWSS}$ [Pa]) and oscillatory shear index ($\mathrm {OSI}$ [-]), both extracted from the dynamic simulations, with the respective percentage differences between static and dynamic simulations ($\Delta \mathrm {TAWSS}$ or $\Delta \mathrm {OSI}$ [%]) for $\mathrm {TAWSS}$ a) and $\mathrm {OSI}$ b) for the healthy control and $\mathrm {TAWSS}$ c) and $\mathrm {OSI}$ d) for the patient; we highlight the positive/negative average values of the differences (blue) and for $\mathrm {OSI}$ only, the binned average based on the $\mathrm {OSI}$ values (orange)

Finally, while we can understand the spatial distribution of $\Delta \mathrm {TAWSS}$ and $\Delta \mathrm {OSI}$ based on the surface plots, they are unable to show the locality of the highest differences between static and dynamic simulations with respect to the range of $\mathrm {TAWSS}$ and $\mathrm {OSI}$. To overcome this, Fig. 5 shows the relationship between the dynamic TAWSS or OSI and the respective percentage difference ($\Delta \mathrm {TAWSS}$ or $\Delta \mathrm {OSI}$ [%]) for HC and P. In addition, we have plotted the average value of $\Delta \phi$ for each of the assessed quantities and a binned average for the $\mathrm {OSI}$. In the case of the binned average, the data were grouped based on a specific range of $\mathrm {OSI}$ values (0.01) for the whole domain and average for the outliers with high $\mathrm {OSI}$ (i.e., when the number of points within a range of OSI was less than 100).

## Discussion

In the present work, we proposed an image-based method for prescribing the motion of the aortic wall for CFD using RBF. The RBF method was chosen since it proved to be a viable approach to represent the complex motion of the aorta. By performing simulations with the predefined motion of the blood vessels, we avoid the necessity of obtaining detailed information regarding the vessel wall (i.e., elasticity and thickness). This information is usually not readily available, making the patient-specific studies challenging for the traditional FSI methods [18–20, 22–24]. Additionally, the computational time of simulations with prescribed motion is comparable with static simulations, as can be seen from our results. This is an important factor, especially considering the clinical applications with larger population studies. We have performed static and dynamic simulations for two geometries: the healthy-control (HC) and patient-specific case (P) with a large TAA located close to the aortic root.

The prescribed RBF-based motion of the thoracic aorta matched well with the 4D-flow MRI, as illustrated in Fig. 1a for mid-acceleration. Some differences could be observed in the proximity of the aortic root. Close to the aortic root, the displacement is also the largest, as visualized in Figs. 1b and 2. In this region (ascending aorta), the motion is rather complex and is a result of the superposition of the axial and radial displacements. The longitudinal displacement (in the feet–head (FH) direction) generated by the physiological strain from the heart, makes an important contribution to the total displacement, as previously reported in several studies on aortic kinematics [13, 14, 30, 31]. It is important to note that this FH-component of aortic motion is not included in the FSI studies of the aorta [18–21], nor in the studies that model the predefined aorta motion based on its wall compliance [22–24], which can have a significant impact on the final results. In contrast to the ascending aorta, the descending aorta is less susceptible to motion due to the presence of the spinal column, and its displacement is predominantly in the radial direction [31], which can be also found in our results; Fig. 2.

The discrepancies between RBF and MRI in the proximity of the aortic root can also be found for the other key-frame geometries; Fig. 1c. The agreement between the original segmentation and RBF, in terms of distance between the surface vertices, is good for most of the investigated aortic domain (as seen in Table 1), except for the root. While this could (potentially) be avoided by locally increasing the density of the control points, the final moving geometry does not have to improve with respect to realistic aortic kinematics. This is caused by the accuracy of the original segmentation, which decreases for key-frames further from the peak systole [32], (Fig. 1c). The segmentation variability can be high, especially close to the root, as shown previously for healthy aortas [33]. Additionally, a similar argument can also be made for including more key-frame geometries. Currently, we only considered four geometries for the proof-of-concept study. This choice was motivated by the segmentation procedure being very time-consuming (approximately four hours per subject), with many manual adjustments necessary. Hence, including more details in the RBF procedure can, on the contrary, increase the amount of uncertainty and degrade our simulations.

Although we have only segmented a limited number of geometries to prescribe the movement, the selected key-frames probably contain the most radial expansion during the cycle. This allows us to capture most of the movement with the least amount of information required. Additionally, the ability of the proposed method to capture the movement based on only a limited number of segmentations is an important advantage for clinical use. In this case, the observer does not need to segment all the phases, saving valuable clinical practice time.

While the limitations behind segmentation of 4D-flow MRI are well-known [32–34], our choice to use this method was motivated by the direct availability of measured flow data. This allows us to obtain accurate inlet and outlet boundary conditions and an estimation of the moving domain from a singular measurement. As shown previously by several studies [35–38] and in Appendix C, velocity profile from measurements should always be imposed as an inlet boundary condition, if available. Additionally, Gallo et al. [39] showed the importance of including as much patient-specific information as possible on all of the outlets of the studied domain to obtain accurate results. By utilizing 4D-flow MRI to obtain all of these, we can create a well-informed, fully patient-specific model of the moving aorta without the necessity of additional measurements on the subjects.

To validate the proposed model, we can directly utilize the 4D flow MRI. As can be seen in Fig. 3, the computed profiles resemble well the ones acquired by MRI, especially in plane 1. More differences can be observed for the other planes, especially for lower velocity values (i.e., further from peak systole). These discrepancies could originate from the computational model, e.g., inaccurate inlet plane for boundary conditions [40] or the choice of outflow boundary conditions [41], which can have an effect up to $5D_i$ upstream, what correlates to the locations of the examined planes. On the other hand, in 4D-flow MRI data, a higher noise-to-signal ratio is present due to the static VENC, for these phases. This can cause limited velocity field acquisition [42] for these phases. Finally, 4D-flow MRI flow has been shown to be consistently underpredicted due to the temporal averaging of the data [43]. However, the simulations generally predict the blood flow behavior in the studied aortas.

Since an increasing number of studies are investigating the effect of aberrant blood flow on the development and rupture of aortic aneurysms, we highlight here the effects of the aorta motion on changes in blood flow patterns. Based on the presented results, certain differences in blood flow patterns were obtained with the static (a rigid wall assumption) and dynamic (predefined aorta motion) simulations; Fig. 3. The latter showed slightly better agreement, qualitatively, with the 4D-flow MRI measurements, especially during the decelerating part of the systole and early diastole. For example, in plane 2 and plane 3 for HC, during mid-deceleration and early diastole, the dynamic simulations were more accurate in capturing the velocity profile measured with 4D flow MRI. On the other hand, the differences between dynamic and static simulations for P are more striking, especially for plane 2 and plane 3, and we cannot make similar conclusions for this case. While these observations are only qualitative, they clearly show the effect of the aortic movement on the flow and the need to examine these differences in a larger number of patients and volunteers.

The flow-derived variables such as the TAWSS and OSI are often used as potential biomarkers to indicate the onset and growth of aortic aneurysm [5]. Elevated regions of WSS were related to more rapid degradation of the extracellular matrix [44]. This causes weakening of the aortic wall (due to lack of elastin), and it was linked to the growth of the TAA [44]. On the other end of the spectrum, low TAWSS may lead to endothelial dysfunction, correlated with thickening of the aortic wall [9, 45] Furthermore, high OSI affects the response of the endothelial cells, and it was connected to the onset of atherosclerosis [46], a condition with high prevalence in patients with aortic aneurysms [47]. Our simulations revealed significant differences between TAWSS and OSI calculated from the static and dynamic simulations for both geometries; Fig. 4. In static simulations, the mean TAWSS is slightly over-predicted, especially in the aortic arch of P; Fig. 4. Nevertheless, the general trends in TAWSS distribution are similar for both of the simulation methods in each studied case. On the other hand, we can observe significantly more differences in OSI, which is consistently underpredicted by the static simulations, especially in the region of interest—ascending aorta; Fig. 4. These observations, including the maximal differences, are in accordance with other studies in the literature that considered the aorta movement—either by FSI [17] or by prescribed motion [24, 25, 29].

Additionally, we observed that aortic wall movement has a considerable effect on the whole range of TAWSS and OSI; Fig. 5. For OSI, it can be seen that the error introduced by the simulations is highest in the regions with low OSI. On the other hand, for higher values of OSI, which are physiologically important [46], the difference between static and dynamic simulation is lower, yet, still as high as 21% for HC and 37% for P. In the case of TAWSS, for which both low and high TAWSS are physiologically important [9, 44, 45], we could not observe such a clear correlation. Here, the difference between static and dynamic simulations is similarly dominant for the whole range of TAWSS (on average, up to 9% for HC and 15% for P, which are similar to other studies in literature [17, 24]). These findings highlight the importance of including aortic wall motion in the simulations, to prevent misinterpretation of the results.

We also found that the differences between static and dynamic CFD for both TAWSS and OSI are correlated with the magnitude of displacement; Fig. 2. The differences in both variables were most significant in the proximity of the aortic root and the ascending part of the aorta, i.e., in the regions where displacement is most prominent. In the arch and descending aorta, the differences between static and dynamic simulations and resulting TAWSS and OSI were smaller. Capellini et al. [29] presented an approach where only a portion of the aorta (ascending thoracic aorta) is considered moving, and the rest of the domain is static. For this case, they showed that the differences downstream of the moving region are negligible. On the contrary, as shown in the presented study, the movement in the arch and descending aorta still affects the flow considerably and should not be omitted. In conclusion, the aspects of the movement of the whole aorta should be included in the new generation of CFD simulations for accurate modeling of blood flow.

Finally, we need to contextualize our findings with respect to other possible sources of uncertainty in the simulations. As shown in our previous study, WSS is highly affected by the segmentation variability, with a local deviation of up to 50% (at peak systole) [33]. Similar or higher uncertainty as found for OSI and TAWSS in our results, was also reported due to inflow rates [48, 49], outflow boundary conditions [39], or inclusion of turbulence modeling [8, 50]. Nevertheless, due to a lack of data for dynamic simulations, specifically for simulations with prescribed motion, it is not possible to generalize whether rigid assumption for the aorta is sufficient in terms of uncertainty, unlike for other parts of the cardiovascular system [51]. For this, a more thorough follow-up study is necessary, including a larger number of pathologies.

Next, we address several limitations of the present work. To demonstrate the proof-of-concept of the adopted RBF-based morphing approach in mimicking the aortic motion, we have considered two geometries: the healthy control and the patient-specific TAA. Future studies can include significantly larger numbers of both subject and patient-specific cases. Moreover, the patient-specific cases should include additional aortic pathologies such as dissection and coarctation [52]. Nevertheless, our work aimed to investigate the feasibility, accuracy, and numerical efficiency of the proposed method. Since we have selected an advanced stage of TAA as one of the test cases, it is expected that the method will also perform well for less-developed pathologies. We also assumed that there was no aortic movement during the diastole. This assumption was a consequence of the unattainable segmentation of the 4D-flow MRI scans due to very low blood flow intensity during this period of the cardiac cycle. However, this assumption is valid since the aortic motion during diastole is limited [16], and we do not expect significant deviations from our findings. Finally, the presented simulation method with aortic motion was coupled with the 4D-flow MRI clinical data; here, we need to address two points: (1) the 4D-flow MRI acquisition is affected by several acquisition parameters such as efficient respiratory motion compensation, VENC, and Sense factor that reflects the amount of parallel imaging for acceleration. All of these can have an effect on the signal-to-noise ratio and hence the segmented data. (2) The current segmentation procedure requires significant manual adjustments to properly capture the exact wall position at particular time instants of the cardiac cycle. We also addressed some of this segmentation variability on the calculated WSS in our previous study [33], where we observed significant variability in WSS due to the segmentation procedure. Since a similar protocol was also used in this study, this could also affect the prescribed wall movement. Additionally, using this technique hinders proper capturing of the aortic dilatation since the absolute difference between the root diameter of the systolic and diastolic phase can be lower than the resolution of 4D-flow MRI, as reported by De Heer et. al. [53]. However, here developed numerical simulation methodology can be directly integrated with other clinical imaging procedures as well (US, MRA, CT), which would improve the segmentation variability and the resolution to capture the motion properly.

## Conclusions

In the present work, we showed how the aortic wall motion can be simulated by applying an efficient image-based geometry morphing approach based on the radial basis function (RBF) interpolation. The simulated aortic motion was in good agreement with the 4D-flow MRI extracted geometries. The developed method proved to be accurate and numerically robust for both considered cases: the healthy-control and the patient-specific aorta with an aneurysm in the aortic root. The computational time for dynamic simulations (with moving aortic walls) was similar to their static (with rigid wall assumption) counterparts, confirming the numerical efficiency of the proposed method. Effects of wall motion in the dynamic simulations were most prominent in the ascending aorta and this improved agreement with the 4D-flow MRI in comparison to the static simulations. We also report on the largest differences between the calculated TAWSS and OSI for static and dynamic simulations in the ascending part of the aorta. This shows the importance and necessity to include aortic wall motion in the CFD simulations in obtaining more accurate flow and flow-derived biomarkers, such as the TAWSS and OSI. Based on here presented proof-of-concept study on two geometries and improved agreement with the 4D-flow MRI, we propose to apply the presented moving wall approach on larger cohorts of patient-specific cases with various aortic pathologies.

## Methods

### Studied cases

Two subjects were included in this study—a healthy control (HC) and a patient (P). The patient had a root aortic aneurysm with a diameter $D=50$ mm and aortic valve regurgitation of 33%. Additional characteristics for both subjects can be found in Table 2.

**Table 2:** Characteristics of the healthy control and patient

		Healthy control	Patient
Gender		Male	Female
Age	[yr]	43	26
Weight	[kg]	85	67
Height	[cm]	195	187
Blood pressure	[mmHg]	115/67	83/46
Mean arterial pressure	[mmHg]	85	63
Heart rate	[bpm]	61	49

### MRI acquisition and data processing

For both subjects, 4D-flow MRI was performed on a 3T MRI system (Elition, Philips Healthcare, Best, The Netherlands) using a hemidiaphragm respiratory navigator with retrospective electrocardiogram gating without echo-planar imaging. Additional parameters in the MRI sequence can be found in Table 3.

**Table 3:** Details of 4D-flow MRI sequence for healthy control and patient

		Healthy control	Patient
Velocity encoding	[cm/s]	150	160
Reconstructed temporal resolution	[ms]	30	38
Echo time	[ms]	2.6	2.7
Repetition time	[ms]	4.5	4.6
Flip angle	[$^{\circ }$]	10	10
Acquired isotropic resolution	[mm]	2.5	2.7
Field of view	[mm^3^]	350 $\times$ 78 $\times$ 160	450 $\times$ 60 $\times$ 150
Turbo field echo factor	[-]	2	2
Parallel imaging factor	[-]	2.5 $\times$ 1.2	2.5 $\times$ 1.2

The acquired 4D-flow MRI data sets were segmented using CAAS MR Solutions v5.2. (Pie Medical Imaging BV, Maastricht, The Netherlands). The protocol for segmentation is identical for both studied subjects. The analysis is initialized by manually placing starting and ending points of the domain at peak systole. The starting point is placed in the aortic root, and the ending points are placed in all major branching arteries of the arch (brachiocephalic trunk, left common carotid artery, and left subclavian artery) and in the abdominal aorta. Subsequently, a 3D volume at peak systole is automatically segmented and manually adjusted (if discrepancies are observed). The manual adjustments for the peak-systolic phase are mostly necessary for the regions with flow recirculation, i.e., in the proximity of the aortic root and downstream the aortic arch. After successful segmentation, the peak-systolic 3D volume is copied to the next phase of interest and manually adjusted for the movement. In this case, the manual interventions are more complex and time-consuming (up to three hours per phase) due to the arterial movement, both caused by the compliance of the aortic wall as well as the movement of the heart. This process is especially time-consuming in the ascending aorta due to its complex movement through the cardiac cycle. The segmentation procedure is then repeated for all of the phases of interest (in total, four instants of the cardiac cycle were extracted—mid-rising systole (point 3 for HC and 2 for P), peak systole (point 6 for HC and 5 for P), mid-decreasing systole (point 9 for HC and 7 for P), and beginning of diastole (point 12 for HC and 10 for P)).

### Geometry pre-processing

**Fig. 6:**
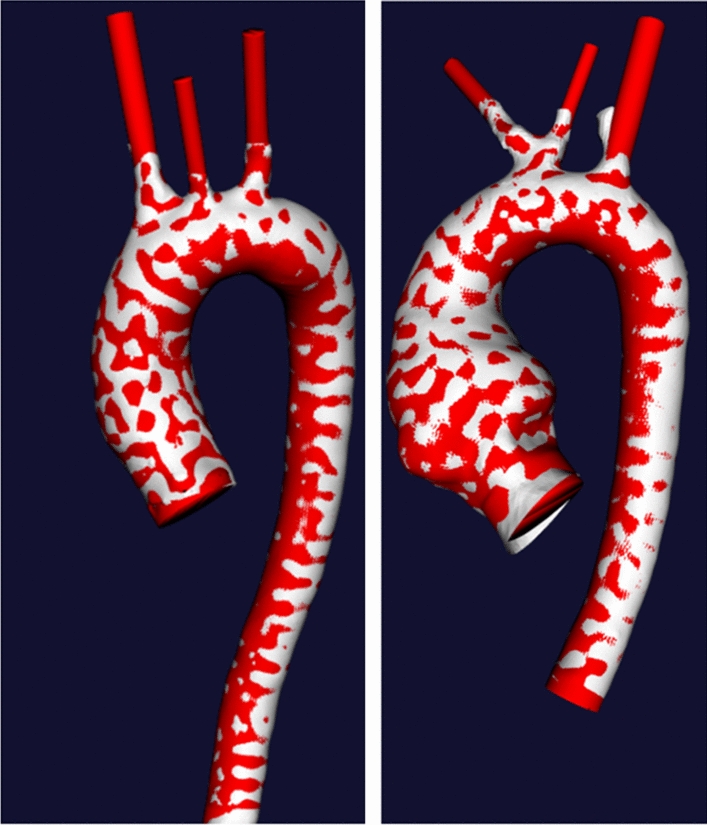
Initial surface obtained from 4D-flow MRI (white) and the geometry after the final step of pre-processing (red) for healthy control (left) and patient (right)

The initial surface obtained via segmentation of 4D-flow MRI is not suitable for CFD due to the relative ’roughness’ of the surface mesh (i.e., variation of the normal vector direction of the segmented surface from its ideal form due to segmentation errors) and inconsistent boundary faces of inlets and outlets. To remove these imperfections, we performed pre-processing of the extracted surfaces using Vascular Modelling Toolkit (VMTK) [54]. The initial (4D-flow MRI) and final surface after pre-processing for peak systole are shown in Fig. 6; note that while the whole aorta was extracted for HC, only the thoracic part was considered for the further simulations and analysis.

To obtain the final surface, the following steps are executed: first, the surface inlet and outlets are cut perpendicular to the arterial centerline. Next, the smoothing step is performed. In this step, the most optimal smoothing should account for the regions with high variation of the normal vectors while preserving the total volume. We have utilized the Taubin smoothing, with pass-band 0.1 and 100 iterations. Compared to other methods, the Taubin smoothing procedure ensures proper smoothing of regions with high variations in surface curvature and avoids extensive shrinkage of the surface [55]. Next, the surface mesh (triangular) is subdivided using the Butterfly method [56] to ensure better surface definition for the computational model. Finally, we added cylindrical extensions on the inlet and outlets in the normal direction of the respective planes. The diameter and the length of the extension are determined based on the diameter of the respective boundary ($D_i$). The length was kept constant for all outlets ($5 \cdot D_i$), and only a very short flow extension was created for the inlet ($0.5\cdot D_i$) to assure the reliability of the applied inlet velocity profile while ensuring the stability of the moving mesh implementation.

### Computational model

**Fig. 7:**
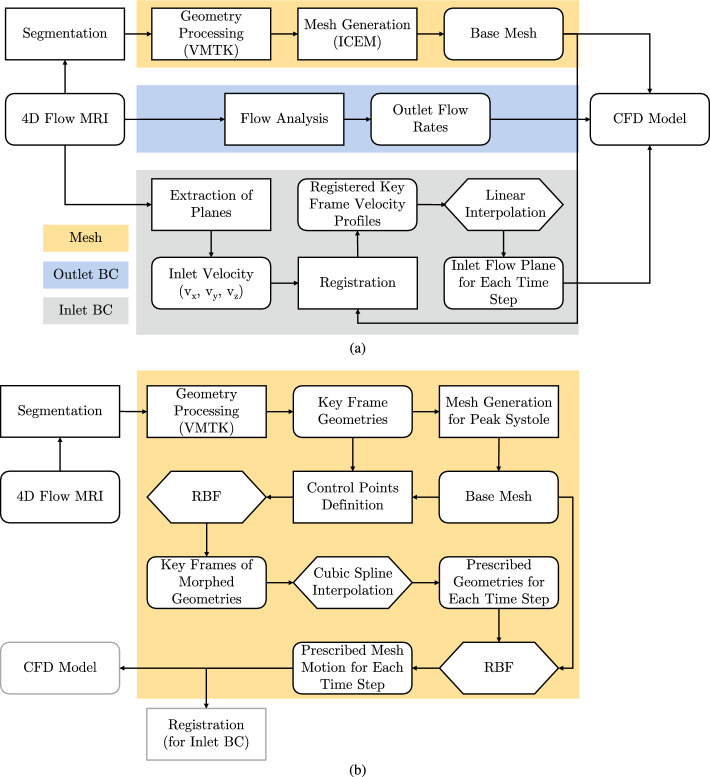
Schematic flow-chart showing the main CFD model inputs (mesh, outlet boundary conditions, and inlet boundary conditions) for the static simulations (**a**) and the details of the dynamic mesh-morphing of the aortic wall for the dynamic simulations (**b**)

The case-specific computational model was developed to take into account the detailed aorta geometry (and its movement), as well as the inlet and boundary conditions (BC) from the 4D-flow MRI scans. The entire algorithm is illustrated in the flow-chart shown in Fig. 7. We have performed simulations with the rigid (static) and moving (dynamic) aortic wall for both subject- and patient-specific geometries. For the latter, additional algorithm details are given in Fig. 7b and will be discussed below.

### Fluid dynamics

In the present work, we adopt the ALE (Arbitrary Lagrangian–Eulerian) formulation for conservation of mass and momentum for a moving numerical mesh, for which the following governing equations are solved [57]:

1 $$\begin{aligned}{} & {} \frac{\partial \rho }{\partial t} + \nabla \cdot \left[ \rho \left( {\textbf {v}} - {\textbf {v}}_g \right) \right] = 0 \end{aligned},$$

2 $$\begin{aligned}{} & {} \frac{\partial \left( \rho {\textbf {v}} \right) }{\partial t} + \nabla \cdot \left[ \rho {\textbf {v}} \left( {\textbf {v}} - {\textbf {v}}_g \right) \right] = -\nabla p + \nabla \cdot \overline{\overline{\tau }}, \end{aligned}$$

where $\rho$ is the fluid density, ${\textbf {v}}$ is the fluid velocity, ${\textbf {v}}_g$ is the grid (or mesh) velocity, *p* is the pressure, and $\overline{\overline{\tau }}$ is the viscous stress tensor $\left( \overline{\overline{\tau }}= \mu \left( \nabla {\textbf {v}} + \nabla {\textbf {v}}^T \right) \right)$, with $\mu$ as the dynamic viscosity of the fluid. Note that for the static simulations (the rigid wall assumption), we have ${\textbf {v}}_g =0$. Additionally, since we employed a moving grid approach for part of our simulations, we need to define the space conservation law as follows:

3 $$\begin{aligned} \frac{d V}{d t} = \int _{\partial V} {\textbf {v}}_g \cdot {\textbf {A}} = \sum _{j}^{n_f} {{\textbf {v}}_{{\textbf {g,j}}}} \cdot {\textbf {A}}_j, \end{aligned}$$

where *dV*/*dt* is the volume derivative of the arbitrary control volume *V*, $\partial V$ is the boundary of the arbitrary control volume *V*, ${\textbf {A}}$ is the face vector area, $n_f$ is the number of faces *j*. Finally, the dot product on the right-hand side is calculated from

4 $$\begin{aligned} {{\textbf {v}}_{{\textbf {g,j}}}} \cdot {\textbf {A}}_j = \frac{\delta V_j}{\Delta t}, \end{aligned}$$

where $\delta V_j$ denotes the volume swept out by the control volume face *j* over each time step $\Delta t$ [58].

Finally, we did not employ any turbulence model and assumed the flow to be laminar. This choice is justified since the mean Reynolds number (*Re*), was lower than the critical *Re* reported for aorta [59] for both of the cases ($Re_{HC}=1890$, $Re_{P}=1480$).

### Boundary and initial conditions

The inlet plane boundary condition was specified as a velocity inlet where all three velocity components at particular instants of the cardiac cycle were extracted from the reconstructed 4D-flow MRI (similarly to other studies [35–38]). This was done using an in-house developed software tool for proper time registration and interpolation of the clinical data. All steps of this procedure are shown in the flow-chart diagram shown in Fig. 7a, and can be summarized as:

1. Using an in-house developed tool for 4D-flow MRI data analysis, all three velocity components ${\textbf {v}} \left( v_x, v_y, v_z \right)$ are extracted from the reconstructed 4D-flow MRI data at the inlet plane of the studied case for each acquired time step (n = 34 for the healthy control, and n = 32 for the patient-specific acquisitions, respectively).
2. Velocity data are linearly interpolated for each (n) and (n+1) time step, where time-step size ($\Delta t$) is based on the requirements of CFD (in the present work, we have $\Delta t$ = 1 ms, for both cases).
3. Inlet (represented by the CFD mesh) is imported from the base mesh (at peak systole), and the interpolated velocity profile is registered on this mesh; for dynamic simulations, the inlet is imported for each time step from the generated moving mesh.
4. The velocity components are then interpreted in the CFD software as a *Profile* and interpolated and projected on the inlet mesh using inverse-distance interpolation.

**Fig. 8:**
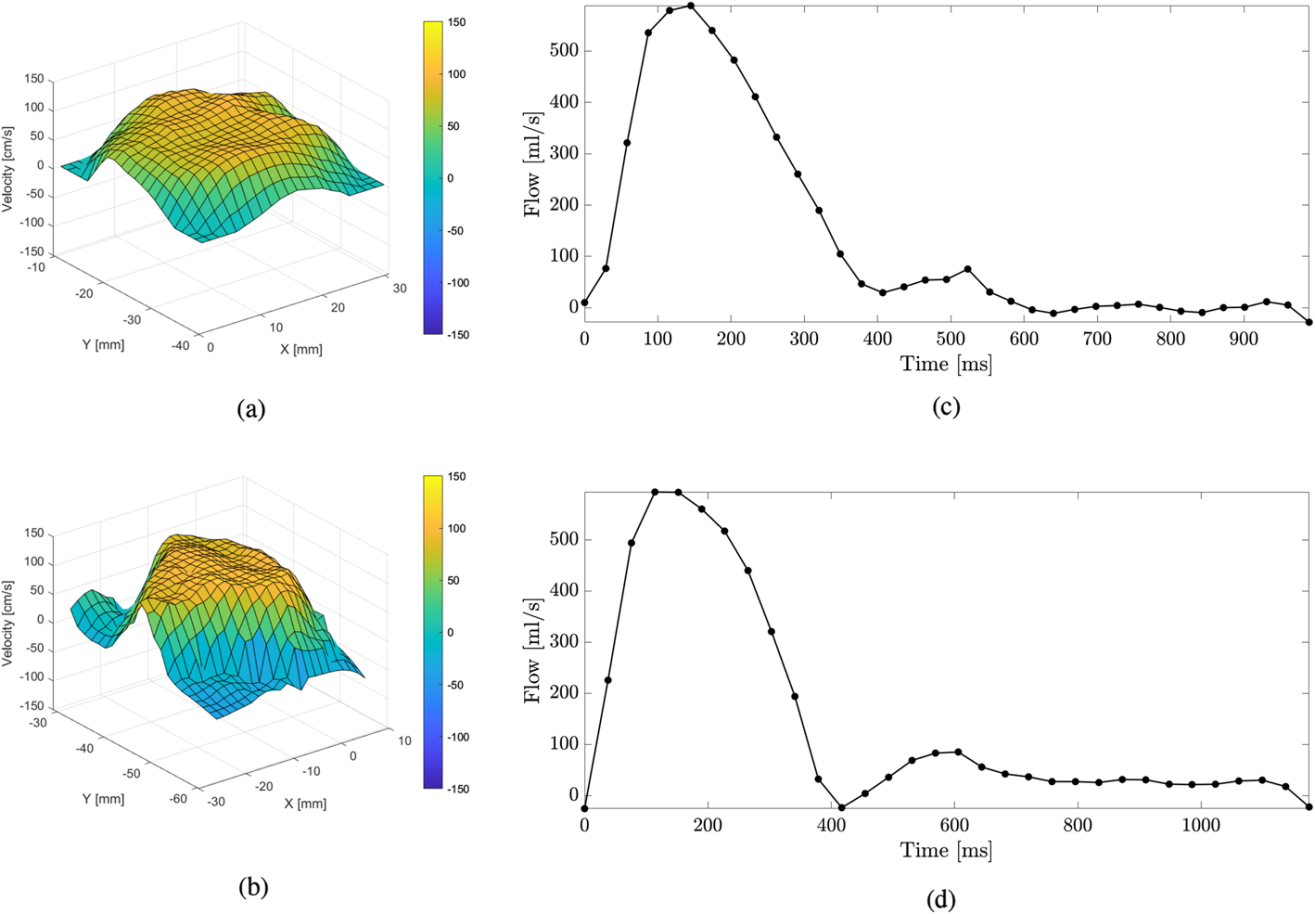
Interpolated inlet velocity profile at peak systole based on 4D-flow MRI for healthy control (**a**) and patient (**b**) and the volumetric flow at the inlet for one cycle for healthy control (**c**) and patient (**d**)

An example of the inlet flow rate and the interpolated velocity profiles at peak systole for the HC and P cases can be seen in Fig. 8.

Outlet boundary conditions were treated as outflow with a predefined fraction of mass flow per outlet. The outflow boundary condition assumes zero-diffusive flux for all flow variables and a mass balance correction at the outlet. The flow fractions at each outlet ($w_N$) were defined based on the 4D-flow MRI measurements. Since the 4D-flow data in the supra-aortic arteries are unreliable due to a low number of voxels, we have exported planes in the upstream and downstream proximity of each bifurcation and, by that, estimated the net flow leaving through each outlet. In addition, due to the presence of bovine arch in P, we have extracted additional planes directly located in the outlets, downstream the brachiocephalic trunk, to account for the flow repartition. Afterward, the fractions at each time step were calculated as follows:

5 $$\begin{aligned} w_n = \frac{Q_n}{Q_i}, \end{aligned}$$

where $w_n$ and $Q_n$ are the flow fractions and the net flow of the respective outlets, $Q_i$ is the net flow at the inlet and *m* is the total number of outlets. Finally, each outlet flow fraction is scaled by the sum of all of the fractions to satisfy ${ \sum _{n=1}^{m} Q_i\cdot w_N }=Q_i$. Then, the scaled fraction at each outlet ($w_N$) is defined as:

6 $$\begin{aligned} w_N = \frac{w_n}{\sum _{n=1}^{m} w_n}. \end{aligned}$$

Applying the measured data at each time-step proved to be more accurate in the definition of the patient-specific simulations, as shown previously by Gallo et al. [39].

The no-slip condition was applied at the wall for both static and dynamic simulations. The definition of wall movement for the dynamic simulations is discussed in detail in the next section. The transient simulations were initialized using the steady-state solution at the peak systole. In total, we have simulated three cardiac cycles to eliminate the influence of initial conditions. We have used only results from the last cardiac cycle for the final analysis.

### Moving wall

In the present study, for dynamic simulations, we have adopted a predefined moving wall approach as shown in Fig. 7b. The wall motion was defined from four key-frame geometries extracted from the 4D-flow MRI (as previously described in MRI acquisition and segmentation section). The full process of the moving mesh generation over the entire cardiac cycle can be summarized as:

1. The 4D-flow-based geometries at key-frames are pre-processed using VMTK (as described in Sec. ) yielding the initial surface of the aorta (in.stl format).
2. For the geometry at the peak systole, various cross-section markers (planes) are introduced to separate the static (branching arteries) and dynamic (the rest of the aorta) segments.
3. The numerical mesh is created for this aortic geometry (base mesh), with a refinement close to the wall.
4. The control points are introduced for the peak systole and all key-frames by the following procedure:
  (i) Control points for the inlet and outlet are defined (circumferential equidistant distribution).
  (ii) A finite number of the planes perpendicular to the flow direction with uniform longitudinal distances are selected; in each of these planes, the radial distances are defined similarly to (i);
  (iii) Additional manually adjusted control points are introduced at locations in the proximity of the branching arteries.
  (iv) The final form of the structured control points matrices are established with $i\times j$ control points (*i* = number of planes, *j* = control points per plane), for HC = 19 $\times$ 6 and P = 18 $\times$ 6.
5. The base mesh (generated in step 3) is morphed using Radial Basis Function (RBF) interpolation of the control points (defined in the previous step), resulting in the morphed surface geometries for all selected key-frames.
6. The key-frames surface geometries are then interpolated in time over the entire cardiac cycle using spline interpolation with smoothing parameter $p=0.999$, resulting in a total of *n *= 1018 and 1212 frames. Note that we assumed no aortic movement during diastole.
7. The generated surface geometries at each time step alongside the base mesh are then used as input for the RBF-based mesh-morphing during the simulations.

**Fig. 9:**
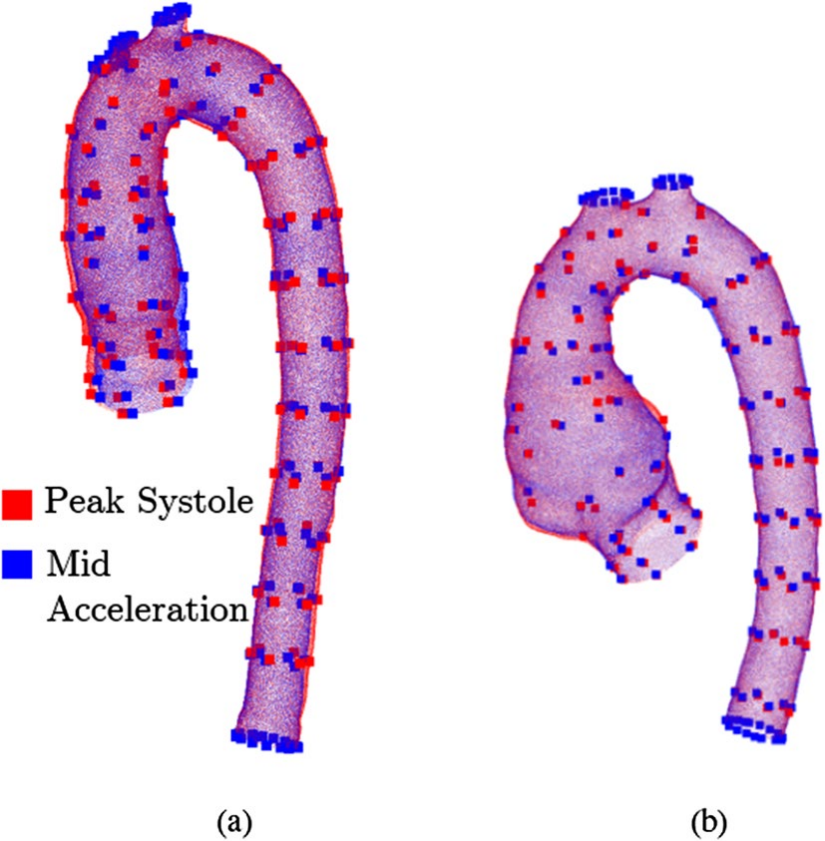
Surface points and control points (big) at peak systole (red) and mid acceleration (blue) for healthy control (**a**) and patient (**b**)

Figure 9 depicts the surface points (both HC and P) for the reference phase (peak systole—red) and for one of the RBF-generated frames (mid acceleration—blue) together with the control points for the two respective phases.

### Physical and solver setup

The blood rheology was accounted for by applying the Carreau–Yasuda model:

7 $$\begin{aligned} \mu _{\text{app}} = \mu _{\infty } + \left( \mu _0 - \mu _{\infty }\right) \left[ 1 + \left( \lambda \dot{\gamma }\right) ^{\alpha }\right] ^{\frac{n-1}{\alpha }}, \end{aligned}$$

where $\mu _{\text{app}}$ is the apparent viscosity, $\mu _{\infty }$ the viscosity at infinite shear, $\mu _0$ the viscosity at zero shear, $\lambda$ the relaxation time, $\dot{\gamma }$ the shear rate, $\alpha$ a shape parameter, and *n* the power-law index. The values for these parameters are adopted from [60], and are $\mu _{\infty } = 2.2$ mPa$\cdot$s, $\mu _0 = 22$ mPa s, $\lambda = 0.110$ s, $\alpha = 0.644$, and $n = 0.392$. The blood density was kept constant ($\rho = 1060$ kg/m$^3$).

The initial mesh was identical for static and dynamic simulations and consisted of tetrahedral elements with refinement close to the wall. We have performed a mesh dependency study for the peak-systolic flow conditions (all details shown in the Appendix). Based on the mesh dependency study, the final mesh consisted of $n=1.58\cdot 10^6$ elements for the HC case and $n=1.47\cdot 10^6$ elements for the P case. Specifically for the dynamic simulations, the smoothing and re-meshing of the 3D mesh were conducted if element skewness was higher than 0.9. We have used the spring-based smoothing with the spring constant factor of one and a maximum of 250 iterations allowed. For re-meshing, the minimal and maximal allowed cell size for the whole domain varied between 1.76 $\times 10^{-4}$ m and 5.76 $\times 10^{-3}$ m.

The simulations were performed using Ansys Fluent 2019 R3 (Ansys, Canonsburg, Pennsylvania, USA). The main computational settings used in this study were: the pressure-based solver, PISO for pressure–velocity coupling, the second-order upwind scheme used for the discretization of convective terms, the second-order central differencing scheme (CDS) used for the discretization of diffusive terms, the time integration was performed by the second-order fully implicit scheme, and the convergence criterion per time step of $10^{-5}$ was used for all quantities.

### Post-processing

The near-wall hemodynamic effects were studied by introducing several quantities averaged over the entire cardiac cycle:

8 $$\begin{aligned} \text{TAWSS} = \frac{1}{T} \int _0^T \left| \overrightarrow{\tau _w}\right| \ \ dt, \end{aligned}$$

where $\mathrm {TAWSS}$ is the time-averaged wall shear stress, *T* is the length of a cardiac cycle, and $\overrightarrow{\tau _w}$ is the wall shear stress,

9 $$\begin{aligned} \text{OSI } = \frac{1}{2}\left( 1-\frac{\left| \int _0^T \overrightarrow{\tau _w}dt\right| }{\int _0^T \left| \overrightarrow{\tau _w}\right| dt}\right) , \end{aligned}$$

where $\mathrm {OSI}$ is the oscillatory shear index. For the dynamic simulations, the values of TAWSS and OSI were projected and visualized on the surface geometry at the peak systole. Additionally, we have calculated the percentage difference ($\Delta \phi$) between CFD_static_ and CFD_dynamic_ for above-defined quantities as:

10 $$\begin{aligned} \Delta \phi =\frac{\phi _{\text {stat}}-\phi _{\text{dyn}}}{0.5(\phi _{\text{stat}}+\phi _{\text{dyn}})} \times 100 \ \ \left( \text{in} \ \ \% \right) , \end{aligned}$$

where $\phi _{\text{stat}}$ and $\phi _{\text{dyn}}$ are the TAWSS or OSI for the static and dynamic CFD simulations, respectively.

## Figures and Tables

**Fig. 10:**
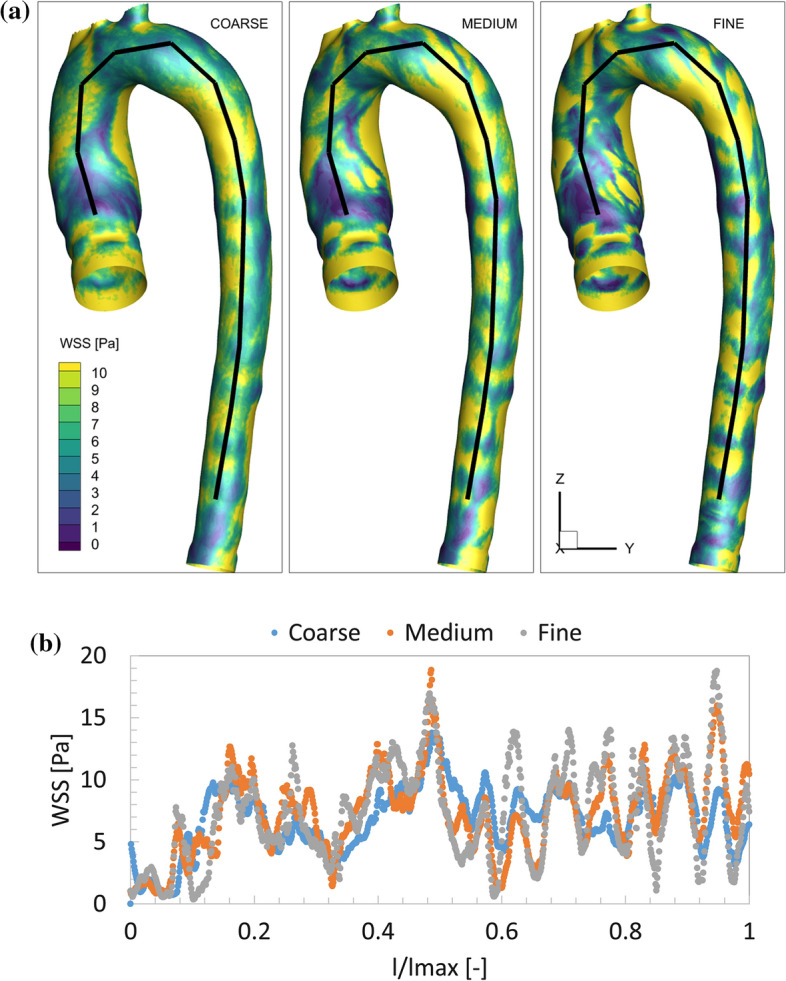
Wall shear stress (WSS [Pa]) for healthy control as obtained for coarse, medium, and fine mesh with visualized extraction line (**a**) and the data extracted alongside the line with normalized line distance ($l/l_{max}$) starting from the root with the respective averaged data (**b**) (coarse—black, medium—orange, fine—grey)

**Fig. 11:**
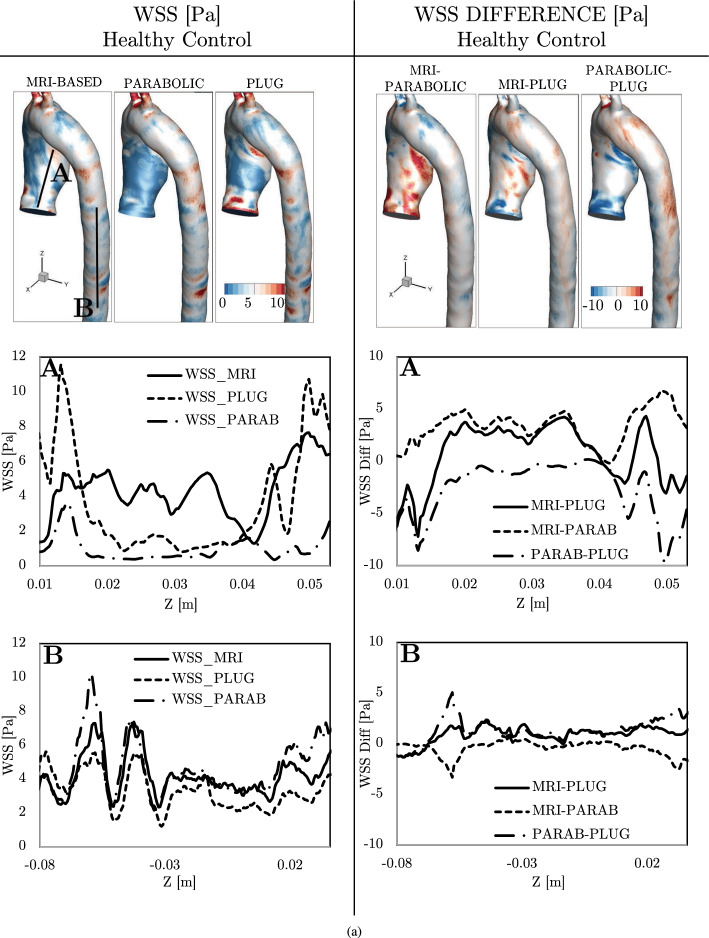
Comparison of wall shear stress (WSS in Pa) from CFD simulations with a varying inlet (MRI-based, parabolic, and plug) for healthy control (**a**) and patient (**b**) and the percentage difference between MRI-based and parabolic, MRI-based and plug, and parabolic and plug. Detailed information was also extracted alongside lines (A—ascending aorta, B—descending aorta)

**Fig. 12:**
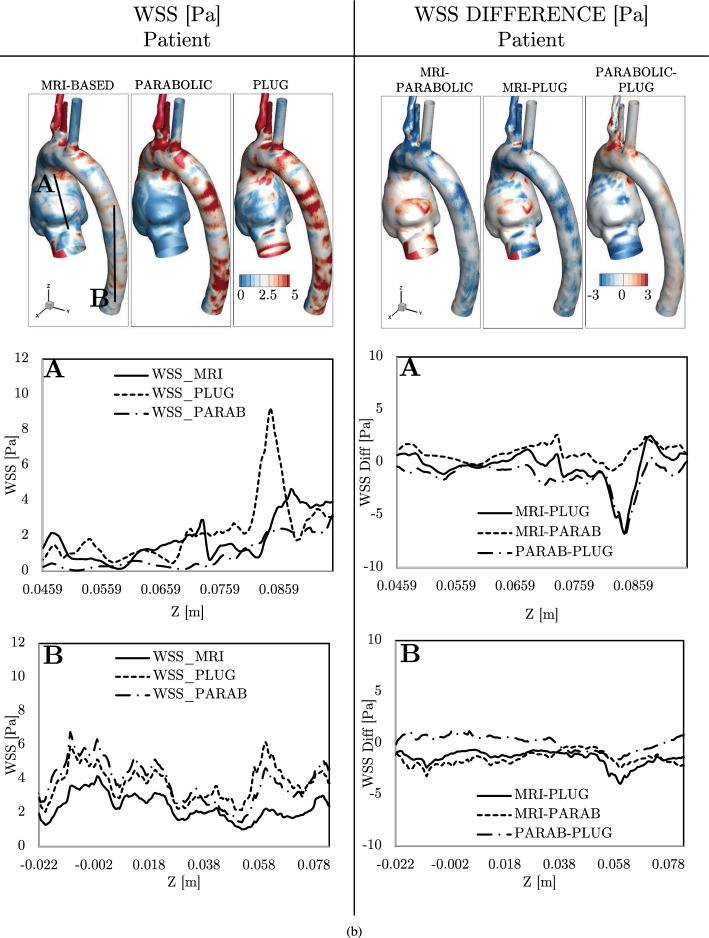
Comparison of wall shear stress (WSS in Pa) from CFD simulations with a varying inlet (MRI-based, parabolic, and plug) for healthy control (**a**) and patient (**b**) and the absolute difference between MRI-based and parabolic, MRI-based and plug, and parabolic and plug. Detailed information was also extracted alongside lines (A—ascending aorta, B—descending aorta)

## Data Availability

The datasets used and/or analyzed during the current study are available from the corresponding author upon reasonable request.

## Appendix A: RBF interpolation—mathematical view

RBF interpolation is based on source points, i.e., the nodal points of the original geometry, and target points which are the nodal points of the deformed geometry. It assumes an unknown smooth function *f* that is given via the set of source and target points. This function is then approximated by an interpolant *s*(*x*) of which the general form is defined as [61, 62]:

11 $$\begin{aligned} s(x)=\sum _{i=1}^N \gamma _i \varphi (||x-x_{k_i}||) + h(x),\quad x \in \mathbb {R}^n, \end{aligned}$$

where *s*(*x*) is the approximating smooth function, *N* the source points, $\gamma _i$ the weights of the radial basis, $\varphi$ the radial basis function, $x_{k_i} = [x_{k_i}, y_{k_i}, z_{k_i}]$ the coordinates of the source points, and *h*(*x*) a polynomial part of which the degree depends on the type of radial basis function used. The polynomial part is added to guarantee the existence and uniqueness of the solution. The parameters $\gamma _i$ and the polynomial coefficients are determined by solving a linear system of equations, with the order equal to *N*. This is defined by the passage- and orthogonality condition [61, 62]:

12 $$\begin{aligned} {\left\{ \begin{array}{ll} s(x_{k_i})=g_{k_i} &{} 1 \le i \le {N} \\ \sum \limits _{i=1}^N \gamma _i q(x_{k_i})=0, \end{array}\right. } \end{aligned}$$

where $g_i$ are known discrete values of displacement of the source points $x_{k_i}$, and *q* polynomials with a degree less than or equal to the degree of polynomial *h*. The first criterion ensures that *s*(*x*) goes through the given values $g_{k_i}$. Secondly, the summation of the product of the weights and the polynomial at the source points $x_{k_i}$ equals zero, which satisfies the orthogonality condition. The values of $\gamma _i$ and polynomial coefficients $\beta _i$ are found by solving [61, 62]

13 $$\begin{aligned} \begin{bmatrix} M_{k_i} &{} H_{k_i} \\ H_{k_i}^T &{} 0 \end{bmatrix} \begin{bmatrix} \gamma \\ \beta \end{bmatrix} = \begin{bmatrix} g_{k_i} \\ 0 \end{bmatrix}, \end{aligned}$$

where $M_{k_i}$ is the interpolation matrix containing the evaluation of the radial function based on the source points, and $H_{k_i}$ the coordinate matrix in which the *i*-th row is $$\big [{\begin{matrix} 1&x_{k_i}&y_{k_i}&z_{k_i} \end{matrix}}\big ]$$. Finally, the displacement of the non-source points $s_m$ is calculated using [61, 62]:

14 $$\begin{aligned} s_m = \begin{bmatrix} M_m&H_m \end{bmatrix} \begin{bmatrix} \gamma \\ \beta \end{bmatrix}, \end{aligned}$$

where $M_m$ is the evaluated matrix based on basis function $M_{mij} = \varphi (||x_{mj}-x_{k_i}||)$, and $H_m$ is the coordinate matrix with *i*-th row $[1 \, x_{m_i} \, y_{m_i} \, z_{m_i} ]$.

We used the multi-quadratics method for the radial basis function, which is defined as:

15 $$\begin{aligned} \varphi (r)=\sqrt{r^2+c^2}, \end{aligned}$$

where *r* is the euclidean distance between the source (*x*) and non-source ($x_{k_i}$) points ($||x-x_{k_i}||$) and *c* is the shape parameter. The shape parameter was estimated based on the mean distance between the source points and their farthest neighbor normalized by the distance to their nearest neighbor. The estimated shape parameters for this study were $c_{HC}=3.2\times 10^{-3}$ for HC and $c_{P}=4.0\times 10^{-3}$ for P.

## Appendix B: Mesh dependency

To investigate the mesh dependency of the simulations, we created three different meshes for HC (coarse—$0.58\times 10^6$, medium—$1.47\times 10^6$, and fine—$4.35 \times 10^6$ control volumes) and performed simulations at the peak-systolic flow conditions. The mesh refinement ratio was $r_{1,2} \approx r_{2,3} \approx 1.40$ for the fine/medium and medium/coarse meshes, respectively. Figure 10 shows WSS for all three meshes and data extracted alongside a line following the aorta for all three meshes.

We have also estimated the Grid Convergence Index (GCI) [63]. The results of the GCI analysis are given in Table 4. Finally, the estimated theoretical value of WSS at zero grid spacing using the Richardson extrapolation to be $f_{h=0} = 7.79$ Pa.

**Table 4:** The total number of elements for mesh sizes coarse, medium, and fine are displayed

Mesh		Coarse	Medium	Fine
# cells	[$\times { {10}}^{{ {6}}}$]	0.56	1.47	4.35
${\text{WSS}}_{{\text{mean}}}$	[Pa]	7.12	7.66	7.77
GCI	[%]	N/A	2.16	0.41

Furthermore, the average WSS over the whole surface is given. GCI is calculated based on the average WSS

## Appendix C: Effect of inlet boundary conditions

To study the possible effects of various inlet boundary conditions (BC) on the flow and WSS distribution we have performed three simulations (for the static aortic wall) with the following velocity profiles in the inlet plane: the MRI-based, parabolic, and plug. The MRI-based inlet was defined using a reconstructed plane at the inlet from the 4D-flow MRI data. The uniform (plug) and parabolic velocity profiles were reconstructed such that their averaged velocity profiles give the corresponding MRI-based inlet flow rate. Using these settings, we have performed simulations for both the healthy control (Fig. 11) and the patient-specific (Fig. 12) geometries and obtained WSS distributions (and differences between the inlet BCs) are shown in Figs. 11 and 12. To make an easier distinction, the range of the WSS contours and particular WSS differences (the color map values) were adjusted per the considered case. Additionally, we have extracted two characteristic profiles along the ascending (line A) and descending (line B) parts of the aortic wall, to analyze the results in detail (Figs. 11 and 12).

Due to the complexity of the moving wall model, the inlet boundary conditions used in the literature are often simplified and defined as either plug or parabolic profiles. In the present work, we have demonstrated that the effects of the various specifications of the inlet velocity profiles were significant in the ascending aorta. In contrast, in descending part of the aorta, the impact of the various inlet velocity profiles was much less significant. Additionally, the quality of the segmentation in the proximity of the aortic inlet can have a significant impact on the calculated WSS distribution.
